# Using a consistency check during data collection to identify invalid responding in an online cannabis screening survey

**DOI:** 10.1186/s12874-022-01556-2

**Published:** 2022-03-13

**Authors:** Christina Schell, Alexandra Godinho, John A. Cunningham

**Affiliations:** 1 Centre for Addiction and Mental Health, Institute of Mental Health and Policy Research, Toronto, Canada; 2 Dalla Lana School of Public Health, University of Toronto, Toronto, Canada; 3 National Addiction Centre, Institute of Psychiatry, Psychology and Neuroscience, Kings College London, London, UK; 4 Department of Psychiatry, University of Toronto, Toronto, Canada

**Keywords:** Cannabis, Invalid responding, Inconsistency

## Abstract

**Background:**

Inconsistent responding is a type of invalid responding, which occurs on self-report surveys and threatens the reliability and validity of study results. This secondary analysis evaluated the utility of identifying inconsistent responses as a real-time, direct method to improve quality during data collection for an Internet-based RCT.

**Methods:**

The cannabis subscale of the Alcohol, Smoking and Substance Involvement Screening Test (ASSIST) was administered as part of eligibility screening for the RCT. Following the consent procedure, the cannabis subscale was repeated during the baseline interview. Responses were automatically compared and individuals with inconsistent responses were screened out.

**Results:**

Nearly half of those initially eligible for the RCT were subsequently screened out for data quality issues (*n* = 626, 45.3%). Between-group bivariate analysis found that those screened out (OUT) were significantly older (OUT = 39.5 years (SD = 13.9), IN = 35.7 years (SD = 12.9), *p* < .001), more had annual incomes less than $20,000CND (OUT = 58.3%, IN = 53.0%, *p* = .047), used cannabis less often in the past 30 days (OUT = 23.3 days (SD = 9.7), IN = 24.8 days (SD = 11.3), *p* < .006), and had lower total ASSIST scores at screener (OUT = 19.3 (SD = 8.0), IN = 23.8 (SD = 10.4), *p* < .001) and baseline (OUT = 17.5 (SD = 7.9), IN = 23.3 (SD = 10.3), *p* < .001) compared to participants who were screened in to the RCT.

**Conclusion:**

Inconsistent responding may occur at high rates in Internet research and direct methods to identify invalid responses are needed. Comparing responses for consistency can be programmed in Internet surveys to automatically screen participants during recruitment and reduce the need for *post-hoc* data cleaning.

## Background

Invalid responding can negatively impact the quality of data collected in self-reported surveys [1]. It occurs when participants answer questions without sufficient effort [2] or attention [3] and has been associated with several factors including distraction [4], fatigue [5], and boredom [1]. Regardless of the underlying causes, during surveys participants can become unmotivated to respond accurately, consistently, or to consider item content or survey instructions [2]. This can result in random data patterns [4] where the participant responds unsystematically and every answer has an equal likelihood of being selected [6]. Non-random response patterns are also possible and may include repeated selection of the same response option (e.g. straightlining) or the use of other discernible patterns (e.g. diagonal lines) [4, 7]. In research, invalid responding may bias validity coefficients, estimated group differences [5], effect size estimates [8], and significance tests [7]. A small amount of invalid responding in a sample (5%) can exaggerate or mute correlations [1], and 10% can create an additional factor in models [1, 9]. It can lower or inflate reliability estimates [1, 9], and impacts internal and external validity [5, 10]. Finally, removing even a small proportion of these cases can improve scale properties [2].

Although there is extensive research on invalid responding, few addictions studies report rates of its occurrence in their samples [6]. This makes it difficult to estimate the overall frequency in addictions research, however, instances as high as 40% have been reported [3, 9]. This is concerning since there is widespread agreement that invalid responding is a serious threat to data quality [1], which can in turn impact research conclusions [7–9] and clinical decisions [5, 10]. While it is likely that most invalid responding is unintentional [4], these responses are nonetheless inaccurate, impact the quality of self-report data, and merit greater attention in addictions research.

It is also important to be aware of the risks of invalid responding in the context of Internet research, especially given the recent proliferation of online studies. For instance, one concern relates to the environment in which the participant completes the survey. Since the participant and researcher are separated, the researcher cannot control possible distractions or monitor participant motivation, attention, or engagement during the survey [1, 11]. Another concern is the possibility of fraudulent respondents [12, 13]. For example, individuals may intentionally misreport on surveys in order to ensure their enrollment in the study or may attempt to re-enroll by adjusting their responses to appear to be different people. Likewise, bots (automatic programs) seek out and complete surveys randomly or inconsistently. Although invalid responding is not unique to Internet administered surveys, there are unique contributing factors, which need to be considered by researchers.

Fortunately, a number of research methods have been developed to try to identify this phenomenon. These procedures can be divided into *post-hoc* data cleaning and direct measures applied during data collection. *Post-hoc* data cleaning is crucial for screening data for invalid responding. Visually inspecting data sets for anomalies such as univariate outliers, out-of-range data, and missing values can help identify suspicious patterns [3]. Frequency curves and norms data are also useful [3], as are more complicated methods such as, testing for multivariate outliers (Mahalanobis D) [1], and measures of internal consistency (i.e. Goldberg’s psychometric antonyms/synonyms, Jackson’s individual reliability index) [3]. Nonsensical answers can also identify suspicious cases (e.g. length of substance use greater than participant age), as can comparing pairs of answers for inconsistent responses [2]. Finally, response times have been used with some success. Shorter completion times are thought to indicate inattention (i.e. a participant does not read the question or consider their response) [7]. However, to varying degrees, these methods can be labour intensive or difficult to implement [1] depending on the type of scales used, the sample size, and survey length [3].

*Post-hoc* methods provide various means of screening data sets for invalid responding, however, deleting data *post-hoc* runs the risk of under powering a sample, altering relationships between variables, or reducing sample diversity [10]. Instead it has been suggested that early detection of invalid responding during data collection might reduce the risk of introducing these other biases [8]. Some more direct procedures have been tested with varying results. Adding bogus items [7] (e.g. “I was born on February 30^th^”), attention checks [7] (e.g. “To show I am paying attention, I will select ‘All the Time’”), and self-reported measure of diligence [1] are popular methods to assess whether participants are reading questions, following directions, and considering their answers.

The following secondary analysis uses data collected during a randomized controlled trial (RCT) of a brief intervention for risky cannabis use. To mitigate concerns related to invalid responding, select responses provided by the individual during the baseline interview were compared to responses provided during the eligibility screener. Those who failed to meet any one of four consistency checks were screened out (not randomized). The objective of this secondary analysis was to understand why individuals were screened out and investigate any differences compared to those who were screened in to the RCT.

## Method

### Participants

Beginning in March 2020, Facebook advertisements recruited individuals living in Canada to participate in a RCT of a brief online intervention for risky cannabis use. The advertisement text invited individuals who were concerned about their cannabis use and “interested in participating in a study to find ways to help people who are worried about their cannabis use,” to complete a brief screener. Individuals were eligible to participate if they were 18 years of age or older, used cannabis in the previous three months, and scored four or more on the ASSIST cannabis scale. Contact information was collected and verified to minimize the risk of bots and fraudulent participants.

### Design

All individuals who met the eligibility criteria and electronically provided informed consent were emailed a personalized link to the baseline survey. The survey link expired two weeks after being sent. The beginning of the baseline survey consisted of the re-administration of the ASSIST cannabis scale. Before continuing with the baseline and receiving the intervention, the quality of the data was assessed by comparing the ASSIST screener and baseline responses.

There were four reasons for which individuals could be screened out for data inconsistencies. First, if they reported not using cannabis and second if their ASSIST total score on the second administration changed to less than four. The RCT was testing a brief intervention for people who use cannabis and a score of four on the ASSIST indicates moderate risk and brief interventions are recommended [14]. Thirdly, an individual was screened out if a response to an ASSIST item changed from being endorsed on the screening survey (e.g. **had** “health, social, legal, or financial problems”) to “never” on the baseline administration (e.g. ‘never’ had “health, social, legal, or financial problems”). Finally, individuals were screened out if their total ASSIST score changed by more than 10 points.

### Alcohol, Smoking and Substance Involvement Screening Test (ASSIST)

The ASSIST is an 8-item tool that was developed by the World Health Organization to screen for substance use and assess risk of harm. The ASSIST assesses several substances including cannabis. The first item determines which substances the respondent has ever used in their lifetime and items 2 to 5 ask about frequency of use and associated risks by using five response options ranging from “Never” to “Daily or Almost Daily.” The score associated with each response varies for each question (“Weekly” is coded 4 for question 2 and 7 for question 5). Item 6 asks if anyone has ever expressed concern about their use and item 7 asks if they have ever tried to cut down or stop using the substance. These two items use three response options: “No, never,” “Yes, in the past 3 months”, “Yes, but not in the past 3 months”. The tool is scored by adding the values for items 2 to 7 and higher scores indicate greater risk. Scores can also be categorized as lower (score of 0 to 3), moderate (score of 4 to 26) and high (score of 27 to 39). Extensive psychometric testing for the ASSIST is still lacking, however, an international study tested the reliability and the validity of the ASSIST in 2002. In this study, the average test–retest reliability coefficient (kappa) for the cannabis subscale was 0.64 and kappas for each item ranged from 0.43 to 0.72. Time between these reliability tests ranged from one to three days. The ASSIST cannabis use scale has a Cronbach’s alpha internal consistency of 0.85 (*n* = 100) [15].

### Ethical approval

The trial protocol was approved by the Centre for Addiction and Mental Health’s standing research ethics review committee.

### Data analysis plan

Bivariate analyses were conducted to investigate possible differences in the demographic data between those who were screened into the RCT or out for data inconsistencies. Likewise, t-tests assessed the difference in continuous variables. Among those who were screened out, frequencies were used to assess which items were subject to data quality issues. All analyses were performed using IBM SPSS Statistics 25.0.

## Results

### Sample

**Fig. 1:**
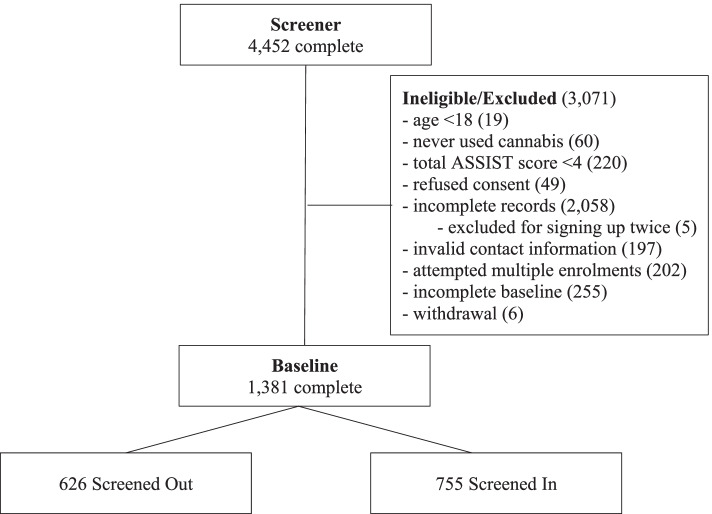
Consort chart

A total of 4,452 individuals completed the screener. Of these, 299 did not meet eligibility criteria (i.e., 18 years of age or older (19); never used cannabis (60); scored less than 4 on the ASSIST (220)). Another 2,304 did not complete the consent process and 207 were excluded for enrolling or trying to enrol multiple times. Additionally, six withdrew and 255 did not complete the second set of ASSIST questions. This resulted in a sample of 1,381 cases where response consistency could be assessed. Of these 626 were screened out and 755 were screened in (Fig. 1).

### Demographics

The average age of the entire sample was 37.5 (SD = 13.5) years, 36.7% (*n* = 507) were male, 45.0% (*n* = 622) were married, 57.8% (*n* = 798) had some post-secondary education, 52.7% (*n* = 728) were employed, and 55.4% (*n* = 765) had an income less than or equal to $20,000 (CND). In order to investigate if there were demographic differences between individuals who were screened in or out, bivariate comparisons were made (Table 1). There were significant differences between the groups with respect to age (mean 39.5 years (SD = 13.9) screened out; 35.7 years (SD = 12.9) screened in, *p* < 0.001). In addition, significant differences were found between the total ASSIST score on the screener (mean 19.3 (SD = 8.0) screened out; 23.8 (SD = 10.4) screened in, *p* < 0.001) and on the baseline scores (mean 17.5 (SD = 7.9) screened out; 23.3 (SD = 10.3) screened in, *p* < 0.001). The groups were also different in the frequency they used cannabis in the past 30 days (mean 23.3 days (SD = 9.7) screened out; 24.8 days (SD = 11.3) screened in; *p* = 0.006).

**Table 1:** Comparisons between those who were Screened In or Out of the RCT due to inconsistencies

Variables	Screened Out (*n *= 626)	Screened In (*n* = 755)	*P*
Male, n (%)	232 (39.0)	275 (39.5)	0.865
Some post-secondary education ﻿, n (%)	347 (55.4)	451 (59.7)	0.107
Married / Common-law﻿, n (%)	290 (46.3)	332 (44.0)	0.382
Full/Part time employed﻿, n (%)	315 (50.3)	413 (54.7)	0.104
Income < = $20,000﻿, n (%)	365 (58.3)	400 (53.0)	**0.047**
Age, mean (SD)	39.5 (13.9)	35.7 (12.9)	** < 0.001**
Total ASSIST screener﻿, mean (SD)	19.3 (8.0)	23.8 (10.4)	** < 0.001**
Total ASSIST baseline﻿, mean (SD)	17.5 (7.9)	23.3 (10.3)	** < 0.001**
Frequency of use, past 30 days﻿, mean (SD)	23.3 (9.7)	24.8 (11.3)	**0.006**

*ASSIST* Alcohol, Smoking and Substance Involvement Screening Test

*SD* Standard Deviation

### Data screening

The average time between the first and second administration of the ASSIST was 2.5 days (SD = 2.75 days). Of the 11 possible data quality checks which were considered inconsistent, 61.8% of the screened out cases were excluded as the result of one data issue, 22.8% because of two, and 15.3% because of three or more (only three participants had more than five data inconsistencies). Table 2 summarizes the data quality issues among those screened out. A change in total ASSIST scores between the screener and baseline resulted in only 86 (13.7%) individuals screened out. The majority of the data quality issues were due to a change on an ASSIST question. A total of 613 (97.9%) participants had at least one inconsistent ASSIST response. Finally, another 20 (3.2%) cases were excluded for indicating on the baseline that they did not use cannabis.

**Table 2:** Change between Screener and Baseline among those Screened Out

Inconsistency Check	Frequency (%) (*n* = 626)
Change in Total ASSIST Scores	
Baseline total score < 4	16 (2.6)
Difference total score of more than ± 10 points	71 (11.3)
Any change in total score	86 (13.7)
Change in ASSIST Item Answers	
3 “strong desire or urge to use”	128 (20.4)
4 use “led to health, social, legal, or financial problems”	215 (34.3)
5 “failed to do what was normally expected because of your use”	209 (33.4)
6 “friend or relative or anyone else ever expressed concern”	151 (24.1)
7 “ever tried to control, cut down or stop using”	177 (28.3)
Any change to ASSIST answers	613 (97.9)
Change in Report of Using Cannabis	
Baseline ASSIST Item 2—Did you use marijuana, cannabis or hashish?	6 (1.0)
Which of the following best captures your pattern of cannabis throughout the week?	14 (2.2)
How many times a day, on a typical weekday/weekend do you use cannabis?	13 (2.1)
How many hours after waking up do you typically first use cannabis?	15 (2.4)
Any change to “never” use	20 (3.2)

*ASSIST* Alcohol, Smoking and Substance Involvement Screening Test

Descriptive statistics of demographic and clinical characteristics of individuals screened out for each type of inconsistency was examined. Cases where the total baseline ASSIST score was less than 4 was combined with those whose scores changed by ± 10 and the results are presented in Table 3 (in the first column, Change in Total ASSIST Score). Change in ASSIST Item Answers describes cases where any ASSIST item response changed to “never” on the baseline. Finally, Change in Report of Using Cannabis consists of cases where on the baseline, the individual reported never using cannabis. Some individuals were screened out for multiple reasons and may be counted more than once.

**Table 3:** Descriptive Characteristics of those who were Screened Out by Type of Inconsistency

Variables	Change in Total ASSIST Score^a^ (*n* = 86)	Change in ASSIST Item Answer^b^ (*n* = 613)	Change in Report of Using Cannabis^c^ (*n* = 20)
Male, % (n)	27.9 (24)	37.2 (228)	30.0 (6)
Some post-secondary education﻿, % (n)	55.8 (48)	55.1 (338)	60.0 (12)
Married / Common-law﻿, % (n)	41.9 (36)	46.5 (285)	40.0 (8)
Full/Part time employed﻿, % (n)	39.5 (34)	50.4 (309)	45.0 (9)
Income < = $20,000﻿, % (n)	59.3 (51)	58.1 (356)	55.0 (11)
Age, mean (SD)	38.0 (13.6)	39.7 (13.9)	34.8 (14.3)
Total ASSIST screener, mean (SD)	21.8 (10.8)	19.3 (7.7)	14.2 (10.8)
Total ASSIST baseline, mean (SD)	15.3 (9.7)	17.5 (7.8)	11.4 (11.6)
Frequency of use, past 30 days, mean (SD)	17.6 (12.2)	23.6 (9.4)	3.6 (9.6)

*Note:* Some individuals were screened out for multiple inconsistencies and may be counted in more than one column

*ASSIST* Alcohol, Smoking and Substance Involvement Screening Test

*SD* Standard Deviation

^a^ Baseline ASSIST total score < 4 and/or change of ± 10 between screener and baseline

^b^ Change to “never” on baseline for any ASSIST item

^c^ Reported never using cannabis on baseline

### Screened in compared to no screening

It is important to consider if the two-step recruitment process meaningfully changed the sample recruited for the RCT. Bivariate comparisons (Table 4) were made comparing the demographics of the sample who were screened in and participated in the RCT and the first 755 individuals who would have been enrolled if there had not been an inconsistency check. There were significant differences between the groups with respect to age (mean 35.7 years (SD = 12.9) screened in, 37.1 years (SD = 13.6) no screening, *p* = 0.040), the total ASSIST score on the screener (mean 23.8 (SD = 10.4) screened in, 22.0 (SD = 9.7) no screening, *p* = 0.001) and on the baseline scores (mean 23.3 (SD = 10.3) screened in, 21.1 (SD = 9.9) no screening, *p* < 0.001).

**Table 4:** Comparison of those Screened In with the Sample that Would have Participated without two-stage screening

Variables	Screened In (*n* = 755)	No Screening (*n* = 755)	*P*
Male﻿, n (%)	275 (39.5)	281 (39.3)	.953
Some post-secondary education﻿, n (%)	451 (59.7)	438 (58.0)	.497
Married / Common-law﻿, n (%)	332 (44.0)	339 (44.9)	.717
Full/Part time employed﻿, n (%)	413 (54.7)	393 (52.1)	.302
Income < = $20,000, n (%)	400 (53.0)	391 (51.8)	.643
Age, mean (SD)	35.7 (12.9)	37.1 (13.6)	**.040**
Total ASSIST screener, mean (SD)	23.8 (10.4)	22.0 (9.7)	**.001**
Total ASSIST baseline, mean (SD)	23.3 (10.3)	21.1 (9.9)	** < .001**
Frequency of use, past 30 days, mean (SD)	24.8 (11.3)	23.9 (9.7)	.068

*ASSIST* Alcohol, Smoking and Substance Involvement Screening Test

## Discussion

To improve data quality and reduce invalid responses for an online survey a real-time comparison of response consistency was trialed during data collection by using two administrations of the ASSIST cannabis scale in an Internet survey. Nearly half (45.3%) of individuals who met initial eligibility criteria were later screened out for inconsistent responses. These individuals tended to be slightly older, used cannabis less frequently in the last 30 days, and had lower total ASSIST scores at screening and baseline compared to those who were screened in. There are many possible explanations underlying these differences including, recall bias or even falsifying data in an attempt to avoid detection. Nonetheless, future research should try to understand whether this is a result of careless responding, recall bias, or due to greater reliability issues of web-based administration and if these vary among different groups.

Without the two-step screening process, the sample would have consisted of 329 (43.5%) participants with inconsistent responses. This sample would have been slightly older with lower total ASSIST scores at screening and baseline. It is possible that the screening process excluded valid respondents and as a secondary analysis, it is not possible to determine how this would have affected the results of the RCT. It was not expected that nearly half of the individuals screened would have inconsistent data, however, if these cases were discarded *post-hoc* during data cleaning, the study would have been severely underpowered jeopardizing the legitimacy of the results. Alternatively, keeping such cases during data cleaning could have had equally devastating impacts by significantly inflating or reducing potential associations between intervention condition assignment and cannabis use outcomes at follow-up [16].

The finding that nearly half of potential participants were screened out is concerning and suggests that invalid responding may occur at a high frequency in Internet surveys and conservative approaches for identifying invalid responding needs to be balanced with the risk of removing valid cases, and Type I /II error [1, 2, 5, 7, 10]. Establishing the point where invalid cases cause more harm than the risk of removing them [9] will be important for future research, however, including at least one measure to identify invalid cases is nonetheless recommended for online surveys [1, 2]. All methods may not measure the same construct [17]. but given the multiple underlying causes for invalid responding, any one method could still improve data quality. A two-step recruitment process, using a consistency check like the one implemented in this study merits further investigation. The ability to program the consistency checks automatically in survey programs is especially useful and provides an alternative to *post-hoc* data cleaning methods.

The advantages of conducting Internet-based survey research has contributed to the increased use of this modality in many fields. Internet surveys are generally affordable, easy to administer, accessible, and can accommodate complicated question skip patterns [10]. Regrettably, Internet survey research is not without its unique challenges. Invalid responding is a recognized data quality issue and screening for this issue needs to become common practice [5] in addictions research. Furthermore, although findings from this study need to be replicated, there may be unexpected benefits to implementing this two-stage method of screening. We speculate that the extra screening undertaken during recruitment may have reduced fraudulent responses and resulted in a more conscientious sample, which subsequently contributed to the high follow-up rates (92% at 3-months; 91% at 6-months) post baseline.

As a secondary analysis, this project was not powered to address invalid responding and its design did not include a control group using another mode of administration. Similarly, the associations discussed here may not generalize beyond Internet surveys of individuals who use cannabis. Another limitation of this analysis is that motivations or reasons underlying the inconsistent responses were not investigated and thus, other explanations or biases may be involved. During development testing of the ASSIST, participants were asked to explain the reasons for their inconsistent responses. In addition to inattention, other common responses included not understanding the question and remembering or forgetting information between testing [15]. While these issues may still contribute to the inconsistent responses, some issues such as question clarity have been improved in subsequent revisions of the ASSIST, including the version used in this study. Nonetheless, we are unable to speculate further as to the source of the invalid responding observed. That is, it is unclear as to whether the inconsistent responding was a limitation of the measure used, the mode of administration (i.e. Internet survey), or a result of participant carelessness or deliberate attempts to be eligible for the study. Whether related to inattention or another variable, inconsistent answers should nonetheless be identified and used with caution in analyses. Finally, time to compete each survey was not recorded, but should be included in future studies as completion time has been associated with invalid responding.

The inconsistent responses observed in this project could be the result of measurement error or an individual making a mistake on a response; however, the inconsistencies could also be related to carelessness, fraudulent responders, or the randomness of bots. Thus, some change in answers is expected between repeated administrations of a tool (e.g., a participant may remember new information). On the ASSIST specifically, such changes can alter the total score by one to four points per question. Placing an upper limit of 10 points on the changes balances the expected measurement error while attempting to reduce the risk of invalid responding. Furthermore, previous research has demonstrated that the web-based measures of the ASSIST has less internal consistency compared to paper-and-pencil administrations [18]. A 10-point difference on the cannabis ASSIST scale demonstrates significant behavioural changes and could potentially have an impact on outcome data [19] and a primary concern for this project was related to the eligibility criteria of moderately risky use. Brief interventions (like the one being tested in the RCT) are recommended for individuals scoring in the moderate range on the ASSIST (4–26). Therefore, it was of particular concern that a 10-point decrease could change a participant from moderate to low risk. Conversely, a 10-point increase was less likely to move a participant from moderate to high risk where a brief intervention would possibly be less beneficial. Nonetheless, future research should systematically investigate the impact of a 10-point cut-off for inconsistencies on the ASSIST.

## Conclusion

Although these findings are limited, the results indicated some demographic differences between those screened in or out of the RCT and differences by type of inconsistency check. Future research should systematically investigate how this type of two-step screening using inconstancies influences data quality, especially due to some of the unique challenges faced by Internet research. Given the results of this study where nearly half of individuals were screened out for a data quality issue, introducing a technique to recognize invalid responding is recommended.

## Data Availability

Data is available from the corresponding author on reasonable request.

## Abbreviations

ASSIST: Alcohol, Smoking and Substance Involvement Screening Test
CND: Canadian Dollar
DFAQ-CU: Daily Sessions, Frequency, Age of Onset, and Quality of Cannabis Use Inventory
RCT: Randomized Controlled Trial
SD: Standard Deviation

## References

[1] Meade A, Craig S (2012). Identifying careless responses in survey data. Psychol Methods.

[2] Huang J, Curran P, Keeney J, Poposki E, DeShon R (2012). Detecting and deterring insufficient effort responding to surveys. J Bus Psychol.

[3] Godinho A, Kushnir V, Cunningham J (2016). Unfaithful findings: Identifying careless responding in addictions research. Addiction.

[4] Johnson J (2005). Ascertaining the validity of individual protocols from web-based personality inventories. J Res Pers.

[5] Conijn J, Franz G, Emons W, deBeurs E, Carlier I (2019). The assessment and impact of careless responding in routine outcome monitoring within mental health care. Multivar Behav Res.

[6] King K, Kim D, McCabe C (2018). Random responses inflate statistical estimates in heavily skewed addictions data. Drug Alcohol Depend.

[7] Leiner D (2019). Too fast, too straight, to weird: non-reactive indicators for meaningless data in internet surveys. Surv Res Methods.

[8] Kountur R (2016). Detecting careless responses to self-reported questionaire. Eurasian J Educ Res.

[9] Fleicher A, Mead A, Huang J (2015). Inattentive responding in MTurk and other online samples. Ind Organ Psychol.

[10] Bauermeister J, Pingel E, Zimmerman M, Couper M, Carballo-Dieguez A, Strecher V (2012). Data quality in HIV/AIDS web-based surveys: handling invalid and suspicious data. Field Methods.

[11] Hardre P, Crowson H, Xie K (2012). Examining contexts-of-use for web-based and paper-based questionnaires. Educ Psychol Measur.

[12] Godinho A, Schell C, Cunningham J (2020). Out damn bot, out: recruiting real people into substance use studies on the internet. Subst Abus.

[13] Bybee S, Cloyes K, Baucom B, Supiano K, Mooney K, Ellington L. Bots and nots: safeguarding online survey research with underrepresented and diverse populations. Psychol Sex. 2021. 10.1080/19419899.2021.1936617. 10.1080/19419899.2021.1936617 PMC9697945 36439051

[14] Humeniuk R, Ali R, Babor T, Farrell M, Formigoni M, Jittiwutikarn J (2008). Validation of the alcohol, smoking and substance involvement screening test (ASSIST). Addiction.

[15] WHO Assist Working Group (2002). The Alcohol, Smoking and Substance Involvement Screening Test (ASSIST): development, reliability and feasibility. Addiction.

[16] Crede M (2010). Random responding as a threat to validity of effect size estimates in correlational research. Educ Psychol Measur.

[17] Stieger S, Reips U-D (2010). What are participants doing while filling out an online questionnaire: a paradata collection tool and an empirical study. Comput Hum Behav.

[18] Khazaal Y, Chatton A, Monney G, Nallet A, Khan R, Zullino D (2015). Internal consistency and measurement equivalence of the cannabis screening questionairre on the paper-and-pencil face-to-face ASSIST versus the online instrument. Subst Abuse Treat Prev Policy.

[19] Moreno G, vanMierlo T (2021). A digital health tool to understand and prevent cannabis-impaired driving among youth: a cross-sectional study of responses to a brief intervention for cannabis use. JMIR Form Res.

